# Role of miR-29b on the regulation of the extracellular matrix in human trabecular meshwork cells under chronic oxidative stress

**Published:** 2009-11-28

**Authors:** Coralia Luna, Guorong Li, Jianmimg Qiu, David L. Epstein, Pedro Gonzalez

**Affiliations:** Department of Ophthalmology, Duke University, Durham, NC

## Abstract

**Purpose:**

To investigate the role of miR-29b on the changes in expression of genes involved in the synthesis and deposition of extracellular matrix (ECM) induced by chronic oxidative stress in human trabecular meshwork cells (HTM).

**Methods:**

Changes in gene expression induced by miR-29b in HTM cells were evaluated by gene array analysis using Affymetrix U133A2 arrays and confirmed by quantitative–PCR. Pathway analysis was conducted using Metacore. Targeting of miR-29b to the 3’-untranslated region of three novel putative targets was evaluated using the Psicheck luciferase system. Chronic oxidative stress was induced by incubation at 40% oxygen for 4–5 days, using cultures incubated at 5% oxygen as controls. Changes in expression in microRNA or gene expression were analyzed by Q-PCR. Cell viability was measured by lactate dehydrogenase release.

**Results:**

Transfection of HTM cells with miR-29b mimic resulted in downregulation of multiple ECM components, including collagens (*COL1A1, COL1A2, COL4A1, COL5A1*, *COL5A2, COL3A1*) *LAMC1,* and *FBN* as well as several genes involved in ECM deposition and remodeling, such as *SPARC*/osteonectin. Three additional genes, *BMP1, ADAM12*, and *NKIRAS2*, were identified as direct targets of miR-29b. Chronic oxidative stress induced a significant downregulation of miR-29b in two HTM cell lines that was associated with increased expression of several ECM genes known to be regulated by miR-29b. The increase in expression of these genes was inhibited by transfection with miR-29b mimic. MiR-29b increased cell viability under both chronic oxidative stress and physiologic oxygen concentrations.

**Conclusions:**

MiR-29b negatively regulates the expression of multiple genes involved in the synthesis and deposition of ECM in trabecular meshwork (TM) cells. Downregulation of miR-29b might contribute to increased expression of several ECM genes under chronic oxidative stress conditions. The balance between the activation of ECM production induced by oxidative stress and the protective effects of miR-29b could be a relevant factor in understanding how oxidative damage may lead to increased deposition of ECM in the TM and contribute to the elevation of intra-ocular pressure in glaucoma.

## Introduction

Chronic oxidative stress has been implicated in both the initiation and progression of pathophysiological phenomena in the trabecular meshwork (TM) in glaucoma [1-3]. Primary open-angle glaucoma (POAG) has been shown to be associated with a signiﬁcant increase in the accumulation of the primary and secondary end products of lipid peroxidation in lipid extracts from TM, aqueous humor, and Schlemm’s canal (SC) [3,4]. Similarly, signiﬁcantly higher levels of the product of DNA oxidation 8-oxo-2,7-dihydro-20-deoxyguanosine have been observed in the TM of glaucoma patients compared with age-matched and sex-matched controls, and the levels of DNA damage were correlated signiﬁcantly with elevation of intra-ocular pressure and visual field defects [1,2,5,6]. Furthermore, reactive oxygen species-mediated damage to the TM has been shown to induce alterations that result in increased aqueous humor outflow resistance [1,7].

Oxidative damage can lead to outflow tissue dysfunction through apoptotic cell loss [8] by inducing certain phenotypic alterations, including those associated with stress-induced senescence. One such phenotypic alteration that has been proposed to explain how oxidative damage might contribute to the malfunction of the TM is the induced upregulation of extracellular matrix (ECM) genes [9]. Such upregulation of ECM-related genes could potentially contribute to the accumulation of ECM components in the sheath-derived plaques (SD-plaques) that have been shown to increase in the TM with aging and in POAG [10]. However, the specific molecular mechanisms involved in the alterations in ECM induced by oxidative stress in TM cells are not completely understood.

MicroRNAs (miRNAs) are an abundant class of noncoding small (~22 nucleotides) RNAs that modulate gene expression at the post-transcriptional level and participate in the regulation of many cellular functions [11-13]. Specifically, miR-29b has been demonstrated to regulate multiple genes coding for ECM proteins, including multiple collagens, fibrillins, and elastin. MiR-29 is a positive regulator of osteoblast differentiation and controls the expression of collagens in differentiated osteoblasts [14]. This miRNA has also been found to be downregulated in various cancers [15-18] and targets extracellular matrix collagens in lung and nasopharyngeal cancer cells [18]. MiR-29b is also downregulated in myocardial infarct, and its downregulation has been shown to contribute to fibrosis in the heart [19]. Thus, miR-29 would be predicted to contribute to the regulation of ECM dynamics in the TM. However, the roles of this miRNA in the TM and the potential involvement on the alterations in ECM synthesis induced by oxidative stress in TM cells have not been investigated.

To gain more insight into the potential role of miR-29b in the TM, we investigated the effects of chronic oxidative stress on the expression of miR-29b, analyzed the changes in gene expression mediated by miR-29b, and evaluated whether alterations in miR-29b expression might alter the effects induced by chronic oxidative stress in human TM (HTM) cells.

## Methods

### Cell cultures and oxidative stress conditions

HTM cell cultures were generated from cadaver eyes, with no history of eye disease, within 48 h post mortem, as previously reported [20]. All procedures involving human tissue were conducted in accordance with the tenets of the Declaration of Helsinski. Cell cultures were maintained at 37 ^o^C in 5% CO_2_ in media (low glucose Dulbecco’s Modified Eagle Medium with L-glutamine, 110 mg/ml sodium pyruvate, 10% fetal bovine serum, 100 µM nonessential amino acids, 100 units/ml penicillin,100 µg/ml streptomycin sulfate, and 0.25 µg/ml amphotericin B). All the reagents were obtained from Invitrogen Corporation (Carlsbad, CA). Oxidative stress was induced by incubation at 40% oxygen 5% CO_2_ for 4–5 days. Cells incubated at 40% oxygen were compared to cells incubated at an oxygen concentration close to the physiological concentration reported in the aqueous humor (oxygen partial pressure PO_2_ 5%) [21].

### Transfections

HTM cells were plated 24 h before transfection with hsa-miR-29b mimic or control mimic (scramble; 20-40pmolar [Dharmacon, Chicago, IL] with Lipofectamine 2000 [Invitrogen] or amaxa nucleofactor kit [Lonza, Basel, Switzerland]) following the manufacturer’s instructions. Briefly, for nucleofection, cells were transfected at density of 4×10^6^ using an endothelial nucleofactor Kit and the program T23 in the Nucleofector, following the basic protocol for primary mammalian endothelial cells and 20 picomolar of miRNAs (Amaxa, scientific-support.US@amaxa.com).  For lipofectamine transfections, cells were seeded at 2-3×10^5^ cells in 12 well plates or 5-6×10^5^ cells in 6 well plates using 40 picomolar miRNAs and 1 or 2 µl of lipofectamine (for 12 and 6 well plates, respectively) and OPTI-MEM I  (Invitrogen). Cotransfections of 293A cells with luciferase 3’untranslated region (UTR) constructs (0.3 µg), miR-29b mimic, or control mimic (20 pmolar) were accomplished using Effectene (Qiagen, Valencia, CA).

### Gene microarray analysis

Gene array analysis was conducted in three independent sets of transfections with either miR-29b mimic or mimic control of the same HTM cell line. Total RNA was extracted three days post transfection using RNeasy kit (Qiagen), amplified (one round amplification) using one cycle target labeling and control reagents (Affymetrix, Santa Clara, CA), and hybridized to human genome U133A2 arrays (Affymetrix) at the Duke University Microarray facility. Raw data were normalized and analyzed using GeneSpring 7.3 (Silicon Genetics, Santa Clara, CA). Genes were filtered to their intensities in the control channel. Pre-mixed polyadenylated prokaryotic sequences from the One cycle target labeling and control reagents kit (Affymetrix) were spiked directly into the samples before target labeling and used as controls. Raw data values below 100 were considered as unreliable. Intensity-dependent normalization was performed per spot and per chip (LOWESS normalization). ANOVA test was performed (p-values ≤0.05 were considered significant) for genes differentially expressed, using the Benjamin and Hochberg False Discovery Rate correction test. The list of genes were compared to three databases that predict targets for miRNAs: Microcosm, TargetScan, and PicTar-Vert. To study the potential biological significance of the changes observed in the arrays, we performed network analysis of differentially expressed genes, using Metacore pathway analysis (GeneGo, St. Joseph, MI).

### Analysis of miR-29b interaction with 3’ untranslated regions

The entire 3’UTRs from bone morphogenic protein 1 (*BMP1*), ADAM metallopeptidase domain 12 *(ADAM12*), and *NFKB* inhibitor-interacting Ras-like protein 2 (*NKIRAS2*) were amplified using primers in Table 1, with carried XhoI and NotI restriction sites in the forward or the reverse position. PCR amplifications from 3’UTRs and the complementary sequences were confirmed by sequencing and cloned into XhoI and NotI sites downstream of Renilla luciferase in the psiCheck2 vector (Promega, Madison, WI). For analysis of luciferase activity, 293A cells were seeded in 12-well culture dishes 24 h before transfection and transfected with psicheck 3’UTR, or the complementary sequence from BMP1, ADAM12, or NKIRAS2 (300 ng), and miRNAs for 29b mimic or control mimic. Luciferase was measured using the Dual Luciferase Kit (Promega, Madison, WI) following manufacturer’s instructions and read in a TD-20/20 luminometer (Turner Designs, Sunnyvale, CA). In this assay, the activities of *Photinus pyralis* and *Renilla reniformis* luciferases are measured sequentially from a single sample. The firefly reporter is measured first by adding Luciferase Assay Reagent II to generate a luminescent signal. After quantifying the firefly luminescence, this reaction is quenched, and the *Renilla *luciferase reaction is simultaneously initiated by adding Stop & Glo® Reagent to the same tube.

**Table 1:** Primers used for amplification of the 3’ untranslated regions of *BMP1*, *ADAM12*, and *NKIRAS2*.

**Gene symbol**	**Forward 5'-3'**	**Reverse 5'-3'**
*BMP1*	GGCTCGAGGGCCTGCCAGGCCTCCCG	GGGCGGCCGCGCAAGAGAAAGGAGCAGGAC
*ADAM12*	GGCTCGAGGTGAAGACAGAAGTTTGCAC	GGGCGGCCGCTCATATCCTCTTATAATTGG
*NKIRAS2*	GGGCTCGAGGCTGCCGTTCCTCTTTCACG	GGGGCGGCCGCGTGTCCAACCAATGCATCAA

### RNA isolation and Quantitative PCR

Total RNA was isolated using an RNeasy kit (Qiagen Inc.) or Trizol (Invitrogen) extraction, according to the manufacturer’s instructions. RNA yields were measured using RiboGreen fluorescent dye (Invitrogen). First-strand cDNA was synthesized from total RNA (1 µg) by reverse transcription using oligodT and Superscript II reverse transcriptase (Invitrogen), according to the manufacturer’s instructions. Quantitative (Q)-PCR reactions were performed in a 20-µl mixture containing 1 µl of the cDNA preparation, 1X iQ SYBR Green Supermix (Bio-Rad, Hercules, CA), using the following PCR parameters: 95 ^°^C for 5 min followed by 50 cycles of 95 ^°^C for 15 s, 65 ^°^C for 15 s and 72 ^°^C for 15 s. β-Actin was used as an internal standard of mRNA expression. The absence of nonspecific products was confirmed by both the analysis of the melt curves and by electrophoresis in 3% Super AcrylAgarose gels (DNA technology, Risskov, Denmark). The primers used for Q-PCR amplification are shown in Table 2. MicroRNAS were extracted using RT^2^ qPCR-Grade miRNA isolation kit (SABiosciences, Frederick, MD) from total RNA extracted with Trizol. miRNAs cDNA (25 ng) were amplified using TaqMan microRNA reverse transcription Kit (Applied Biosystems, Foster City, CA) and specific primers for miR-29b and U6B (Applied Biosystems), as a standard. Q-PCR products were amplified using TaqMan® Universal PCR Master Mix (Applied Biosystems), following manufacturer’s instructions. Briefly, TaqMan quantification is a two step process, in the reverse transcription step cDNA was reverse transcribed from RNA enriched with miRNAs using miR29b and U6B specific primers. PCR products were amplified from these cDNAs using 29b and U6b probes and the recommended PCR parameters: 95 ^°^C for 10 min followed by 40 cycles of 95 ^°^C for 15 s and 60 ^°^C for 1 min. During PCR, the probe anneals specifically to a complementary sequence between the forward and reverse primer sites, when the probe is intact the proximity of the reporter dye to the quencher dye results in suppression of reporter fluorescence. The DNA polymerase cleaves only probes that are hybridized to the target, the cleavage separate the reporter from the quencher and this separation results in increased fluorescence by the reporter. The fluorescence threshold value (C_t_) was calculated using the iCycle system software (Bio-Rad). The results were expressed as mean value± SE (standard error) in three independent experiments.

**Table 2:** Primers used for Q-PCR amplification.

**Gene symbol**	**FORWARD 5'-3'**	**REVERSE 5'-3'**
*STC1*	TCAGAGACAGCCTGATGGAG	CCTCACCTCGGAGGTTCCTG
*STC2*	TCTGCACCTCGGCCATCCAG	TCAGAATACTCAGACTGTTC
*COL4A1*	CCTGGCTTGAAAAACAGCTC	AGGCCTAGTGGTCCGAATCT
*CRABP2*	GTGATGCTGAGGAAGATTGC	CCACAGTCTGCTCCTCAAAC
*TIMP3*	TGCAGCTGGTACCGAGGATG	CAGGCACTAATTTCATTGTC
*LOXL2*	TACCTGGAGGACCGGCCCATG	AGGTCATAGTGGGTGAACAC
*COL1A1*	AGCCAGCAGATCGAGAACAT	TCTTGTCCTTGGGGTTCTTG
*COL1A2*	TGCAAGAACAGCATTGCATAC	GGCAGGCGAGATGGCTTATTTGTT
*COL3A1*	CCATGAATGGTGGTTTTCAG	GTGTTTAGTGCAACCATC
*COL5A1*	GGCTGTGCTACCAAGAAAGG	GAGGTCACGAGGTTGCTCT
*COL5A2*	TGATCCTGAGACTCTTGAAG	GGTGGTCATTGTCATTGGTC
*LAMC1-1*	AATGAAGCCAAGAAGCAGGA	ATGGACAGCAGCAGAGGAGT
*SPARC*	CCGGGACTTCGAGAAGAACT	CTCATCCAGGGCAATGTACT
*FBN1*	CTGCAAGAGGATGGAAGGAG	GGTAAATCCGGGAGGACATT
*BMP1*	GTGTGGCCCGATGGGGTCAT	CCCGCAAGGTCGATAGGTGAA
*ADAM12*	ATGTGGAAAAATCCAGTGTC	GCAAGCACAAGCCCTGGGTC
*NKIRAS2*	CAGGAGCAGCGGCGTGTAGA	GCCATCCAAGGAGCCGCTGC
*ACTB*	CCTCGCCTTTGCCGATCCG	GCCGGAGCCGTTGTCGACG

### Cell viability assay

To evaluate changes in cell viability induced by miR-29b, cells were transfected with control mimic or miR-29b mimic. Cell viability was assayed after 5 days at 40% and 5% oxygen, by measuring the lactate dehydrogenase released to the culture media as a result of plasma membrane damage using the Cito Tox 96® Non-Radioactive Cytotoxicity assay (Promega) following the manufacturers instructions. Briefly, LDH was measured in culture supernatants and in cell lysates from each well, in a 30-min coupled enzymatic reaction, which results in the conversion of a tetrazolium salt (INT) into a red formazan product. The amount of color formed is proportional to the number of lysed cells. LDH in the supernatant was corrected for the total LDH (Supernatant/Supernatant + cell lysis). For each sample the quantification was performed in duplicate. The results were expressed as mean value±SE in three independent experiments.

## Results

### Changes in gene expression induced by miR-29b in human trabecular meshwork cells

Differences in gene expression induced by miR-29b were evaluated by gene array analysis using Affymetrix U133A2 chips. HTM cells were transfected with miR-29b mimic and gene expression was compared to that in cell cultures transfected with a control mimic. One hundred sixteen genes represented by 181 probes were significantly (p≤0.05) upregulated or downregulated more than 1.5-fold. Thirty-one percent of these transcripts were predicted in at least one of the three miRNA databases as putative targets for miR-29b. MiR-29b downregulated several ECM structural proteins, as collagens (*COL5A2, COL5A1, COL4A1, COL3A1, COL1A1*, and *COL1A2*) laminin C, fibrillin 1, and microfibrillar-associated protein 3; and extracellular matrix regulators, such as *MMP14*, *LOXL2, SERPINH1, SPARC, TNFAIP6*, and *ADAM 12*. Other matrix regulators, such as plasminogen activator inhibitor 2, *RECK,* and *TIMP3,* showed upregulation. Table 3 shows transcripts upregulated or downregulated by more than twofold and some selected genes related to ECM that were significantly downregulated or upregulated between 1.5- and 1.9-fold.

**Table 3:** Genes up-or down-regulated after transfection with hsa-miR-29b in HTM cells.

**Genebank**	**Symbol**	**p value**	**Fold**	**Microcosm**	**Targetscan**	**Pictar**
**Selected genes up or down regulated by 2 fold or greater**						
**Down-regulated**						
AF130082	*COL3A1*	0.0062	-4.310	*	*	*
AI983428	*COL5A1*	0.0065	-3.876			
N30339	*COL5A1*	0.0036	-3.597			
NM_000090	*COL3A1*	0.0068	-3.571	*	*	*
BC000658	*STC2*	0.0284	-3.356			
K01228	*COL1A1*	0.0154	-3.3		*	*
Y15916	*COL1A1*	0.0036	-3.268		*	*
AI300520	*STC1*	0.0177	-3.236			
AL575735	*COL5A2*	0.0036	-3.096		*	*
AI743621	*COL1A1*	0.0188	-3.086		*	*
W46291	*ADAM12*	0.0121	-3.077		*	
BE251211	*LOXL2*	0.0177	-3.03			
U05598	*AKR1C2*	0.0008	-2.959			
M33376	*AKR1C2*	0.0068	-2.915			
AW188198	*TNFAIP6*	0.0447	-2.817			
U46768	*STC1*	0.0275	-2.817			
AU144167	*COL3A1*	0.0068	-2.786	*	*	*
AL575735	*COL5A2*	0.0093	-2.695		*	*
AF117949	*LOXL2*	0.0163	-2.475			
NM_024089	*KDELC1*	0.0395	-2.463	*	*	*
NM_006186	*NR4A2*	0.0144	-2.445			
NM_007115	*TNFAIP6*	0.0144	-2.398			
NM_001353	*AKR1C1*	0.0079	-2.398			
NM_003155	*STC1*	0.0062	-2.375			
BE962749	*PPIC*	0.0036	-2.347	*	*	*
NM_003474	*ADAM12*	0.0342	-2.32		*	
AF118094	*TAF11*	0.0163	-2.232	*		
AL050136	*TMF1*	0.0072	-2.222			
S68290	*AKR1C1*	0.0065	-2.222			
AA530892	*DUSP1*	0.0236	-2.155			
AI922605	*COL4A1*	0.0157	-2.155	*	*	*
NM_004353	*SERPINH1*	0.0068	-2.146			
BC001131	*HIST1H2BG*	0.0263	-2.137			
NM_005689	*ABCB6*	0.0121	-2.123	*	*	*
NM_000943	*PPIC*	0.0275	-2.114	*	*	*
AL567376	*LYPD1*	0.0132	-2.058			
NM_000089	*COL1A2*	0.0181	-2.045	*	*	*
NM_006455	*SC65*	0.043	-2.004			
BC000055	*FSTL1*	0.0067	-2		*	*
**Up-regulated**						
NM_022817	*PER2*	0.0144	2.01			
NM_021245	*MYOZ1*	0.0144	2.354			
NM_006895	*HNMT*	0.0068	2.442			
NM_001878	*CRABP2*	0.0163	2.607			
**Selected genes up or down regulated between 1.5 and 1.9 fold**						
**Down-regulated**						
NM_004995	*MMP14*	0.0154	-1.953			
Z48481	*MMP14*	0.0275	-1.88			
NM_002293	*LAMC1*	0.0143	-1.689	*	*	*
BE222709	*MFAP3*	0.0063	-1.543	*	*	*
AL575922	*SPARC*	0.0062	-1.529		*	*
NM_000138	*FBN1*	0.0128	-1.529	*	*	*
**Up-regulated**						
NM_002575	*SERPINB2*	0.05	1.9			
NM_000362	*TIMP3*	0.0461	1.83			
NM_002160	*TNC*	0.0157	1.74			
NM_021111	*RECK*	0.0068	1.503			

This table shows genes that significantly change expression after transfection with miR-29b compared to controls by gene array analysis. The asterisks indicates the miRNA databases that predicts these genes as putative targets for miR29b.

To validate Affymetrix microarray data, changes in expression of 14 genes were analyzed by Q-PCR in three independent HTM cell lines different from the one used for array hybridization (Table 4).

**Table 4:** Validation of gene expression changes after transfection with hsa-miR29b.

**Gene symbol**	**Arrays**		**HTM -1**		**HTM-2**		**HTM-3**	
	**Fold**	**p-value**	**Fold**	**p-value**	**Fold**	**p-value**	**Fold**	**p-value**
*COL1A2*	-2.0450	0.0181	-1.5874	0.0086	-2.2974	0.0006	-2.1189	0.0104
*COL5A2*	-3.0960	0.0036	-2.7007	0.0013	-2.0946	0.0486	-1.9543	0.0410
*COL5A1*	-3.8760	0.0065	-1.8234	0.0095	-2.6390	0.0138	-2.4340	0.0017
*COL3A1*	-4.3100	0.0062	-3.0314	0.0167	-2.9622	0.0002	-2.1685	0.0088
*COL1A1*	-3.3003	0.0154	-1.7818	0.0019	-2.8945	0.0000	-2.0467	0.0416
*COL4A1*	-2.1552	0.0157	-2.5247	0.0078	-2.3217	0.0091	-1.4473	0.0042
*STC1*	-3.2362	0.0177	-1.4641	0.0132	1.0353	0.3437	-1.6818	0.0349
*LOXL2*	-2.4752	0.0163	-1.8234	0.0718	-1.7818	0.0044	-2.1936	0.0493
*STC2*	-3.3557	0.0284	-2.2191	0.0221	-1.4811	0.0029	-1.8234	0.0243
*FBN1*	-1.5291	0.0128	-1.6434	0.0015	-2.1435	0.0053	-1.6434	0.0500
*LAMC1*	-1.6892	0.0143	-1.7211	0.0184	-2.2449	0.0072	-1.9770	0.0136
*SPARC*	-1.5291	0.0062	-1.4641	0.0464	-2.5787	0.0066	-1.4983	0.0219
*CRABP2*	2.6070	0.0163	1.8446	0.0222	2.8284	0.0109	2.0467	0.0251
*TIMP3*	1.8300	0.0461	2.7638	0.0258	-1.3044	0.3918	1.9000	0.0311

Fold changes refers to the ratio of the expression values of the cells transfected with miR-29b over the cells transfected with scramble. P-values for the Q-PCR refer to the t-test between normalized C_T_ values in cells transfected with miR-29b versus cells transfected with scramble.

### Functional network analysis of gene expression changes induced by miR-29b

**Figure 1:**
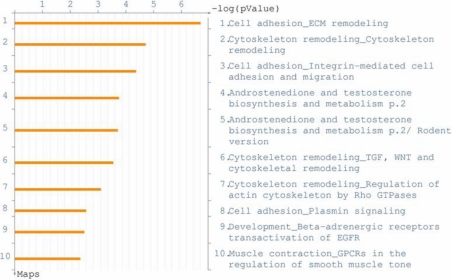
Pathway analysis of changes in gene expression induced by miR-29b. Genes showing changes in expression higher than 1.5 fold (p<0.05) after transfection with miR-29b mimic using Affymetrix U133A2 arrays were analyzed with Metacore pathway analysis. The canonical pathway maps used in this analysis represent a set of 650 signaling and metabolic maps generated from the GeneGo database (GeneGo,St. Joseph, MI). The figure shows the 10 canonical pathways most significantly affected by transfection with miR-29b mimic compared to controls. In the figure, ECM represents extracellular matrix; TGF represents transforming growth factor; EGFR represents epidermal growth factor; and GPCRs represents G protein-coupled receptors.

**Figure 2:**
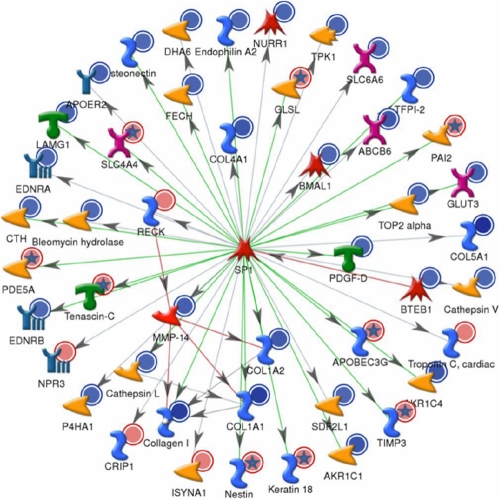
Genes known to be regulated by SP1 that showed significant differences in expression after transfection with miR-29b mimic by array analysis. Metacore analysis of the genes showing significant differences (p<0.5) in expression in the Affymetrix U133A2 arrays identified SP1 as the transcription factor more significantly (p= 4.82 E-106) associated with these gene expression changes. Green lines represent upregulation by SP1, red lines a downregulation, and gray lines an unspecified effect. Genes significantly upregulated in gene array analysis of cells transfected with miR-29b are labeled with red dots. Genes significantly downregulated by miR-29b are labeled with blue dots. Inconsistencies between the array data and the effects predicted by Metacore based on the literature are labeled with a star.

In order to identify the pathways and regulatory elements more likely associated with the changes in gene expression induced by miR-29b, genes significantly (p<0.5) upregulated or downregulated by 1.5-fold in the array analysis were further analyzed using MetaCore algorithms. The three canonical pathways most significantly affected by miR-29b are represented in Figure 1 and include cell adhesion–ECM remodeling (p=1.5 E-107); cytoskeleton remodeling (p=2 E-105), and cell adhesion–integrin-mediated cell adhesion and migration (p=4 E-105; Figure 1). Analysis of transcription factor regulation identified SP1 as the transcription factor most significantly (p=4.82 E-106) involved in the regulation of genes affected by miR-29b with 46 nodes (Figure 2).

### Targeting of the 3’ untranslated regions of *BMP1*, *ADAM12*, and *NKIRAS2* mRNA by miR-29b

**Figure 3:**
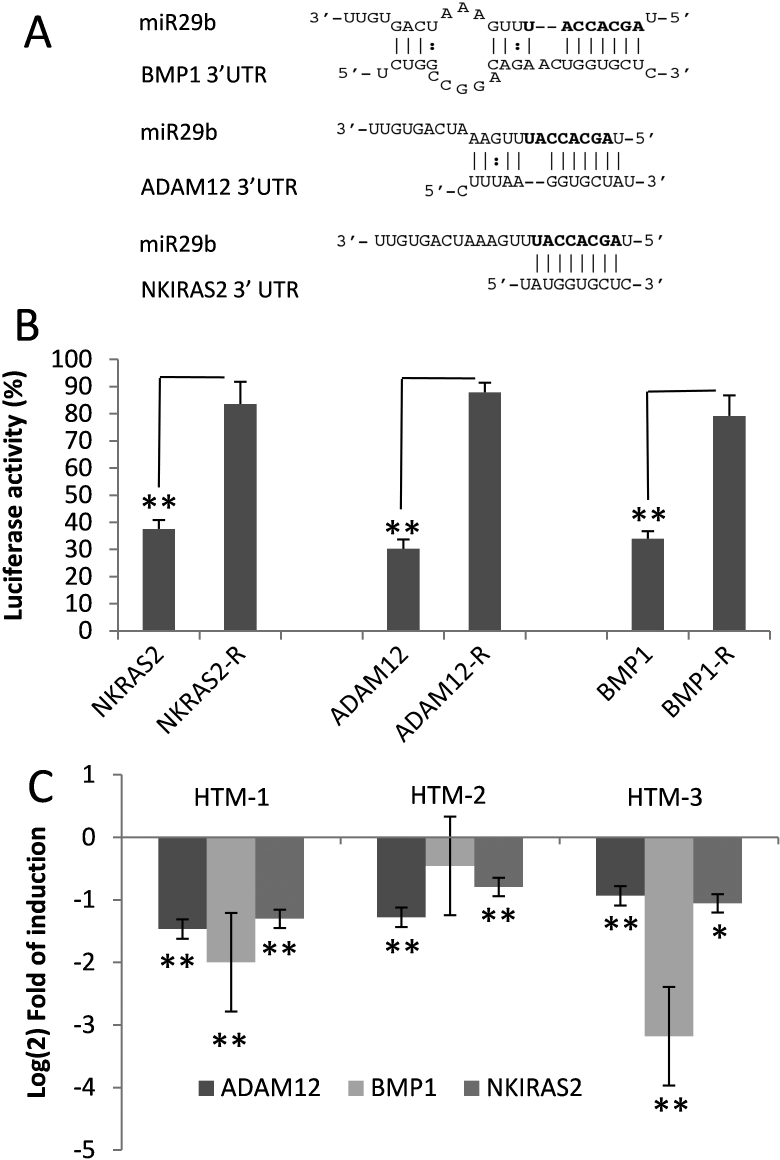
Targeting of the  3’-unstranslated regions of *BMP1*, *ADAM12*, and *NKIRAS2* by miR-29b. **A**: Predicted interactions between miR-29b with the 3’-unstranslated region (3’ UTR) of *BMP1* (PicTar-Vert), *ADAM12* (TargetScan), and *NKRAS2* (PicTar-Vert). Seed regions are highlighted in bold. **B**: Luciferase activity in 293 cells cotransfected with psicheck vectors containing the 3’UTR or complementary sequence (R) from *BMP1*, *ADAM12*, or *NKIRAS2* and miR-29b or scramble. **C**: Changes in expression of *ADAM12*, *BMP1*, and *NKIRAS2* were measured by Q-PCR after transfection with miR-29b mimic or scramble. The figures represent the logarithm of the fold change in gene expression compared to cells transfected with scramble in three different cells lines. Bars represent standard error from three different experiments; one asterisk indicates a p≤0.05, and two asterisks indicate a p≤0.01.

Computational predictions indicate that miR-29b shares complementarity with sequences in the 3’UTR of three genes found to be downregulated by miR-29b according to the gene array analysis: *BMP1, ADAM12*, and *NKIRAS2* (Figure 3A). We investigated whether miR-29b could interact with the 3’UTRs of these genes by using the psiCheck2 luciferase assay system. MiR-29b mimic significantly reduced luciferase expression in cells cotransfected with the 3’UTR of *BMP1*, *ADAM12*, or *NKIRAS2* compared to mimic control (scramble). The decrease in luciferase activity was completely or significantly prevented when the 3’UTR complementary sequences were used (Figure 3B). Downregulation of these gene transcripts by miR-29b was confirmed by Q-PCR in three HTM cells lines (Figure 3C).

### Effects of chronic oxidative stress on the expression of miR-29b.

**Figure 4:**
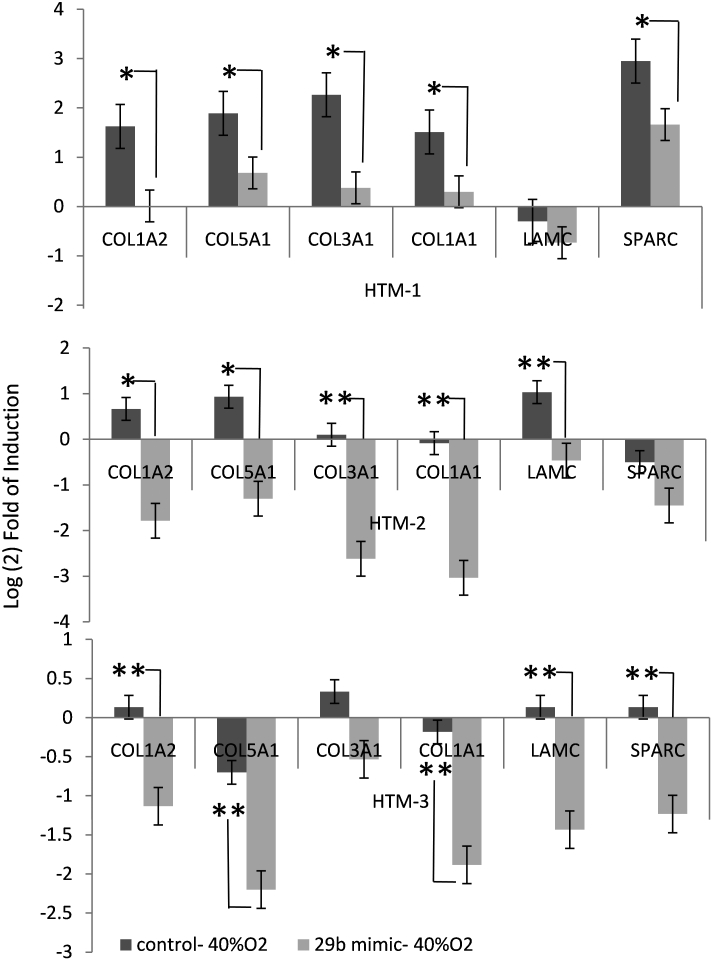
Changes in miR-29b induced by chronic oxidative stress. To investigate if miR-29b changes with chronic oxidative stress, three human trabecular meshwork (HTM) cell lines were incubated during 4 days at 40% oxygen, and the changes in the expression of miR-29b were quantified by quantitative–PCR (Q-PCR) and compared to nonstressed controls incubated at 5% oxygen. The figures represent the logarithm of the fold change in gene expression between cells incubated at 40% oxygen compared to controls. Bars represent standard error from three different measurements. One asterisk means p≤0.05.

Changes in expression of miR-29b induced by chronic oxidative stress were analyzed by Q-PCR in three independent HTM lines after 4 days at 40% oxygen compared to parallel cultures incubated at 5% oxygen. In these conditions miR-29b decreased significantly between 2- and 2.5-fold in two of the three cell lines analyzed and showed no significant change in a third line (Figure 4).

### Role of miR-29b on changes in expression of extracellular matrix genes induced by chronic oxidative stress.

**Figure 5:**
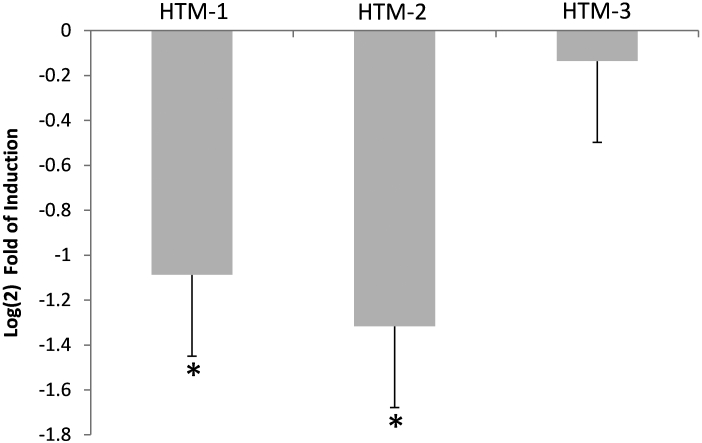
The role of miR-29b on changes in expression of extracellular matrix genes induced by chronic oxidative stress. To investigate whether miR-29b under chronic oxidative stress conditions could affect the changes in expression of several extracellular matrix (ECM) genes, three human trabecular meshwork (HTM) cell lines were transfected with miR-29b mimic or control mimic and incubated under oxidative stress conditions (40% O_2_) for 4 days. The changes in expression of *COL1A2, COL5A1, COL3A1, COL1A1, LAMC1*, and *SPARC* compared to nonstressed controls incubated at 5% oxygen and transfected with control mimic were quantified by quantitative-PCR (Q- PCR). The figures represent the logarithm of the fold change in gene expression between cells incubated at 40% oxygen transfected with either miR-29b mimic or mimic control compared to control cultures (5% oxygen, mimic control) for three individual cell lines. Bars represent standard error from three different experiments. One asterisk means p≤0.05, and two asterisks mean p≤0.01.

To investigate whether the downregulation of miR-29b observed in two cell lines could mediate alterations in gene expression induced by chronic oxidative stress, the effects of incubation at 40% oxygen on the expression of six genes known to be regulated by miR-29b (*COL1A2*, *COL5A1*, *COL3A1*, *COL1A1*, *LAMC*, and *SPARC*) were analyzed in the same three HTM cell lines used in the previous experiment. The experiments were conducted in cells transfected with either control mimic or miR-29b mimic. Although there was a high level of variability in the effects of chronic oxidative stress on the expression of the selected genes, the two cell lines where miR-29b had been found to be downregulated more than twofold under oxidative stress conditions showed a significant increase in the expression of several genes regulated by miR-29b. In contrast, incubation at 40% oxygen had little effect on the expression of these genes in the cell line where miR-29b had not been found to be altered by chronic oxidative stress. In all cell lines, transfection with miR-29b mimic led to either a significant downregulation or decrease in the upregulation mediated by chronic oxidative stress of all genes analyzed compared to cultures transfected with control mimic (Figure 5).

### Effects of miR-29b on cytotoxicity

**Figure 6:**
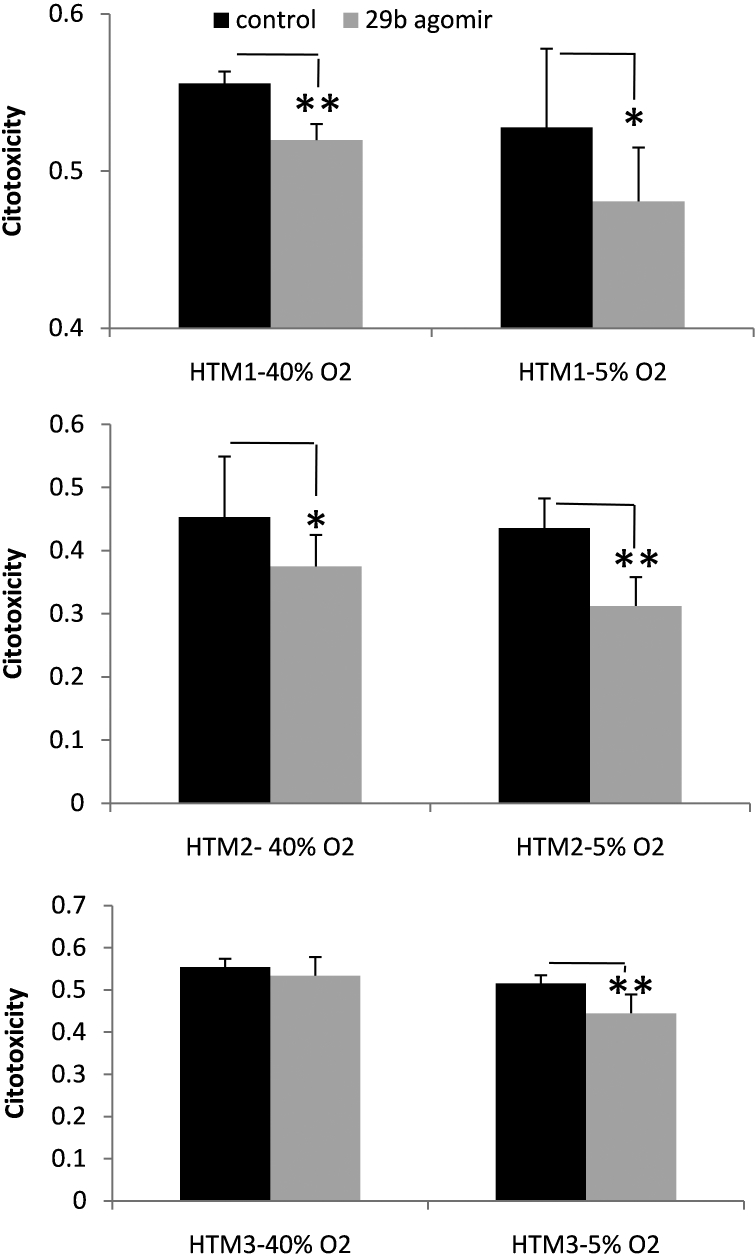
Effects of miR-29b on the cytotoxicity of human trabecular meshwork  (HTM) cells. HTM cells transfected with miR-29b mimic showed significantly lower levels of cytotoxicity measured by lactate dehydrogenase release compared to cells transfected with control mimic when incubated at both 40% oxygen and 5% oxygen conditions. Bars represent standard error from three different experiments. One asterisk means p≤0.05, and two asterisks mean p≤0.01.

HTM cells transfected with control mimic or miR-29b mimic were subjected to 40% or 5% oxygen and analyzed for cytotoxicity after 5 days. Cells transfected with miR-29b mimic showed a significant decrease in cytotoxicity compared to the control in both oxygen concentrations (5 and 40%) except for HTM cell line 3 at 40% O_2_, which showed a nonsignificant decrease in cytotoxicity (Figure 6).

## Discussion

Our results showed that miR-29b negatively regulates the expression in HTM cells of multiple genes involved in ECM synthesis, deposition, and remodeling. In addition, incubation under chronic oxidative stress conditions (4 days, 40% oxygen) resulted in a significant downregulation of miR-29b in two of the three HTM cell lines analyzed. This downregulation was associated with an increase in the expression of several ECM genes known to be regulated by miR-29b. The upregulation of these genes by chronic oxidative stress was inhibited by transfection with miR-29b mimic.

The multiple effects on the expression of ECM components observed in HTM cells were consistent with the antifibrotic activity previously reported for miR-29b in the heart [19]. These effects include the downregulation of validated targets, such as *COL1A1*, *COL1A2*, *COL3A1*, *FBN1*, and *SPARC* [19,22].

MiR-29b also downregulated numerous genes that have not been confirmed as direct targets of this miRNA. Some of these genes contained sequences in their 3’UTRs that are predicted to anneal to miR-29b and may potentially interact with miR-29b. Among these genes, *BMP1*, *ADAM12*, and *NKIRAS2* were confirmed by luciferase analysis to contain 3’UTRs that can be directly targeted by miR-29b and should be considered targets of this miRNA. However, many of the gene expression changes induced by miR-29b affected genes that lack any predicted targeting sequence for miR-29b and appear to be secondary targets. Pathway analysis indicated that a number of these genes are positively regulated by the transcription factor SP1, which is a validated target of miR-29b [23], suggesting that inhibition of SP1 by miR-29 may be an important factor in the overall effects on gene expression mediated by miR-29b.

Exposure to chronic oxidative stress conditions for 4 days resulted in a significant decrease in expression of miR-29b in two (HTM1 and HTM2) of the three cell lines analyzed. Interestingly, while these two cell lines showed increased expression of several genes known to be validated targets of miR-29b, the only cell line where miR-29b was not downregulated (HTM3) showed no significant increase in the expression of these genes. The upregulation of ECM genes mediated by chronic oxidative stress in cells lines HTM1 and HTM2 was inhibited by transfection with miR-29b. These results suggest that downregulation of miR-29b could be a mechanism that mediates some of the alterations in ECM induced by chronic oxidative stress. The variability observed in the levels of upregulation of each gene analyzed between the two cell lines where miR-29b was significantly downregulated is likely to result from the influence of multiple pathways involved in the regulation of the ECM under oxidative stress conditions. However, our results suggest that miR-29b may be an important regulatory component of this process.

The genes selected for this study included important components of the ECM, such as *COL1A2*, *COL5A1*, *COL3A1*, *COL1A1*, *LAMC*, and the matricellular protein *SPARC* that is known to promote ECM deposition [24,25]. Increased expression of these genes could potentially influence the physiology of the outflow pathway by contributing to increased deposition of collagen and other ECM components in the TM [26-30]. Therefore, the variability observed in the effects of chronic oxidative stress on the expression of miR-29b could be relevant to understanding individual differences in susceptibility to pathophysiological alterations induced by chronic oxidative stress in the outflow pathway.

In addition to changes in the expression of ECM components, our results also showed that miR-29b had a protective effect and decreased cell death. Such effect on cell viability contrasts with the pro-apoptotic effects reported for this miRNA in other cell types [16,31,32]. Members of the miR-29 family, including miR-29b, have been demonstrated to activate *p53* by targeting *p85α* and *CDC42* [31]. Because of the known pro-apoptotic effects of *p53*, this function of miR-29b would initially be expected to increase apoptosis under chronic oxidative stress conditions. However, under mild stress conditions, p53 is also known to exert antioxidant and survival effects that are believed to be aimed at preventing oxidative damage and ensuring the survival and repair of cells encountering only low levels of damage [33]. Under the chronic oxidative conditions used in our model, cells might suffer only a moderated level of damage, which would lead to a pro-survival function of *p53*. Furthermore, transient expression of miR-29a, which shares the same seed region of miR-29b and also regulates p53 through targeting of p85α and *CDC42*, did not result in increased apoptosis in osteoblasts [31]. Thus, the pro-apoptotic effects reported for miR-29 may be dependent on the cell type or the specific stress conditions affecting the cells. It is also possible that additional targets of miR-29b that have not been characterized may be involved in the effects of miR-29b on cell survival in HTM cells. For instance, one of the new targets identified in this study, *NKRAS2*, is a negative modulator of *NFKB*, and its downregulation by miR-29b could potentially facilitate the anti-apoptotic effects of *NFKB*.

In conclusion, miR-29b negatively modulated the expression of collagens and other key components of the ECM in TM cells and decreased cytotoxicity in the presence of chronic oxidative stress. The downregulation of miR-29b observed in two cell lines could contribute to some of the alterations in ECM metabolism and cell viability mediated by chronic oxidative stress in HTM cells. The balance between the activation of ECM production induced by oxidative stress and the protective effects of miR-29b could be a relevant factor in understanding how oxidative damage may lead to increased deposition of ECM and decreased cellularity in the outflow pathway and contribute to the elevation of intra-ocular pressure in glaucoma. Strategies to increase miR-29 expression in TM cells may be beneficial to limit ECM deposition, prevent cell loss, and maintain normal levels of aqueous humor outflow facility.
